# The modeling of attraction characteristics regarding passenger flow in urban rail transit network based on field theory

**DOI:** 10.1371/journal.pone.0184131

**Published:** 2017-09-01

**Authors:** Man Li, Yanhui Wang, Limin Jia

**Affiliations:** 1 State Key Laboratory of Rail Traffic Control and Safety, Beijing Jiaotong University, Beijing, China; 2 School of traffic and transportation, Beijing Jiaotong University, Beijing, China; 3 Beijing Research Center of Urban Traffic Information Sensing and Service Technology, Beijing Jiaotong University, Beijing, China; Beihang University, CHINA

## Abstract

Aimed at the complicated problems of attraction characteristics regarding passenger flow in urban rail transit network, the concept of the gravity field of passenger flow is proposed in this paper. We establish the computation methods of field strength and potential energy to reveal the potential attraction relationship among stations from the perspective of the collection and distribution of passenger flow and the topology of network. As for the computation methods of field strength, an optimum path concept is proposed to define betweenness centrality parameter. Regarding the computation of potential energy, Compound Simpson’s Rule Formula is applied to get a solution to the function. Taking No. 10 Beijing Subway as a practical example, an analysis of simulation and verification is conducted, and the results shows in the following ways. Firstly, the bigger field strength value between two stations is, the stronger passenger flow attraction is, and the greater probability of the formation of the largest passenger flow of section is. Secondly, there is the greatest passenger flow volume and circulation capacity between two zones of high potential energy.

## Introduction

Urban rail transit(URT) has become people’s first choice due to its convenience, efficiency and safety. A networked operation in URT has taken place in some international metropolis such as Beijing, Shanghai, and Tokyo, with the scale of passenger flow increasing and a congestion forming in a continual way, especially in rush hours. Taking Beijing as an example, the subway lines has reached 19 by the end of 2016, with the mileage of 574km. The increasing correlation among stations and lines has led to an increasing complexity in the characteristics of passenger flow change. Currently, 4 large stations, including Dawanglu Station, Xierqi Station, Dongzhimen Station, and Beijing Railway Station, are more liable to form congestion. The reasons are that these stations have served as transfer ones and meanwhile there are usually apartment buildings, office buildings and shopping malls around them.

Thus, how to conduct an effective research into the attraction relationship and the space-time distribution regarding passenger flow among stations, has become an urgent issue, the solution of which will render a support in project planning and network operation management.

The internal interaction and potential attraction of passenger flow among stations in the network is a relatively complicated phenomenon, **with the main existing problems** in this research as follows.

1. With respect to the representation of the space-time distribution characteristics regarding the passenger flow in the network, the indirect method has been used in the traditional research to carry out an analysis of the changing trend of the passenger flow [1–3]. Some scholars have utilized decaying models of the road performance to establish the distribution model [4].
  Passenger flow distribution is a result of passenger route choice decision-making. Referring to passenger route choices, train delays has been considered as an influence factor [5–7]. Moreover, the analysis of passenger travel behaviors is considerably important [8]. Zhu (2011) [9] studied a scenario-based route choice model and calculation method against the background of the 2010 World Expo in Shanghai, China. Some research has been conducted regarding to passenger flow patterns analysis based on smart card data [10–13], passenger flow distribution [14–16], congestion evolution under interrupted operation [17,18], and passenger flow forecasting [19–20].
  These researches have some limitations as it depends on the changes of the distance between the stations and the city center. However, there is no uniqueness of the city center any more, with the deepening of urbanization and increasing passenger flow.
2. In terms of the analysis of characteristics of URT networks [21–26], most research has been focused on the network degree, the complexity and robustness of metro networks [27–31], betweenness centrality [32–35]. While in the definition of betweenness centrality, it has been assumed that the weights of all the edges are equal (the value is 1). However, for most actual cases of networks with additional flows, such as those in logistics, road transport, and subway, we can’t just assume the equality of all the edges.
3. With the application of the field theory to the logistics field [36,37], mainly including its concept, basic characteristic quantity, etc., relevant scholars have conducted a research into the internal laws of logistic operation system. The application of the field theory to the transportation field [38–41] mainly involves the modeling analysis of the effect of passage congestion and diffusion, the space distribution characteristics of the flow and the framework of passenger flow.
  What the field theory describes is about the distribution of physical quantity in an area of some given space realm, with all the elements having their own specific distribution laws and interactions among them. However, there is not much research into the systematic characterization of the internal potential attraction and interaction regarding passenger flow.

In view of what is mentioned above, this paper has made a quantization of the passenger flow attraction, with the introducing field theory to reveal the internal characteristics and operation mechanism in respect to passenger flow distribution. **The contributions made in this research are as follows.**

1. For the purpose of a comprehensive analysis of the potential attraction and space-time distribution characteristics of passenger flow, this paper has introduced a new concept of the gravity field of passenger flow and proposed the computation methods of field strength and potential energy from the perspective of topological attributes and flow attributes of the network.
2. This paper offers a computation method of betweenness centrality based on the optimum path as the key parameter of field strength, with the simultaneous verification of the optimum path through the employment of OD matrix. In order for the computation of potential energy, we have applied Compound Simpson’s Rule Formula to get a solution to the function.

The structure of this paper is as follows. Part One focuses on the construction of the model of the network, the concept of gravity field, field strength and potential energy, as well as the relevant methods of computation involving the parameters of field strength, the definition of the betweenness centrality of optimum path, the parameter computation of potential energy function based on Compound Simpson’s Rule Formula. Part Two deals with the simulation and verification of all theories in Part One, including the source and background of data, the determination and verification of related parameters in Part One and the force analysis of the spatial linkage of passenger flow.

## Concepts and methods

### 1. The modelling of URT network

**Fig 1:**
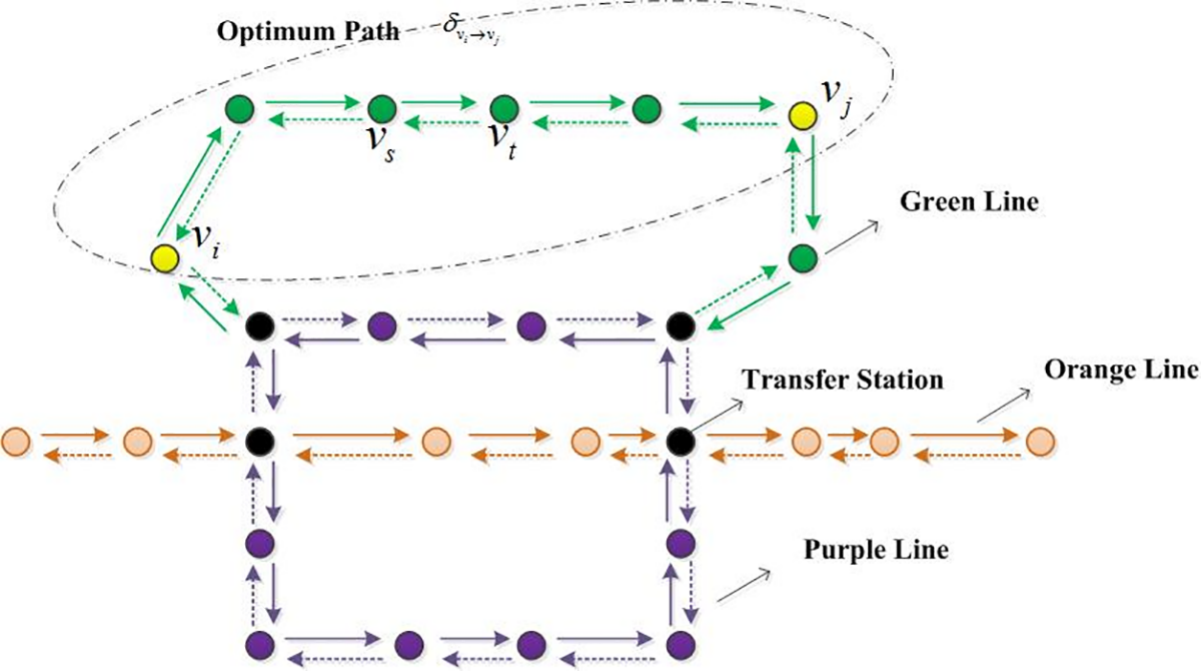
The pattern of topological structure of the URT network.

The URT network consists of some operational lines which are composed of some stations and zones among stations. Taking station *i* as node *v*_*i*_ and zones among stations *i*,*j* as edge *l*_*ij*_ = {*v*_*i*_ → *v*_*j*_}, considering the operational directions of the trains, we construct the pattern of topological structure of URT network as is shown in **Fig 1**.

**Definition 1 neighbors:** As for any node *v*_*i*_,*v*_*j*_, if there is an accessible path between them, *v*_*i*_ and *v*_*j*_ are defined as neighbors. *r*_*ij*_ represents the distance, with the calculation of the minimum numbers of stations between *v*_*i*_ and *v*_*j*_.

**Definition 2 optimum path:** It refers to the path with the smallest weight of $w_{e_{st}}$ between the two nodes of *v*_*i*_ and *v*_*j*_ in the network, with the mathematical formula as follows.

$$\delta_{v_{i}\to v_{j}}=\min\;\sum w_{e_{st}}$$ (1)

$\delta_{v_{i}\to v_{j}}$ is the impedance function of the path.

In the URT network, $\delta_{v_{i}\to v_{j}}$ is two-tuples of travel distance and transfer times. For the convenience of computation, the travel distance is replaced by the train operation time among the zones, with the omission of time for the passengers to get on and off the train. The mathematical formula is as follows.

$$\sum w_{e_{st}}=\sum_{\Gamma_{v_{i}\to v_{j}}}T_{v_{i}\to v_{j}}+\kappa \sum_{\Gamma_{v_{i}\to v_{j}}}T_{v_{tr_{i}}}$$ (2)

Where $\Gamma_{v_{i}\to v_{j}}$ stands for all the stations attached to the path of *δ* between the node-pairs OD. $T_{tr_{i}}$ stands for the time of the *i*th transfer and $T_{v_{i}\to v_{j}}$ refers to the operation time of the train according to the timetable. *κ* is used to stand for the traveling impedance coefficient of the transfer times.

**Definition 3 The betweenness centrality *BC***_***i***_ **of the optimum path:** *d*_*jk*_ is set as the total number of the optimum path between the *v*_*j*_ and *v*_*k*_ and *d*_*jk*_(*v*_*i*_) is the total number of the optimum path when the node of *v*_*j*_ travels through the node of *v*_*i*_ to the node of *v*_*k*_, i.e., ∀*j*,*k* ∈ *G* and *j* ≠ *k*. If the optimum path travels through the node of *v*_*i*_, we have *d*_*jk*_(*v*_*i*_) = 1, otherwise *d*_*jk*_(*v*_*i*_) = 0. The formula of *BC*_*i*_ is

$$BC_{i}=\frac{\sum_{j,k\in G,j\neq k}d_{jk}\left(v_{i}\right)}{\sum_{j,k\in G,j\neq k}d_{jk}}$$ (3)

The betweenness centrality of the optimum path regarding the station reflects the degree of topological significance of node *v*_*i*_, a large value represents its high capacity to attract passenger flow in the network.

**Definition 4 The source and gathering point of passenger flow:** As for the node of *v*_*i*_ in the network, the classification is made from the perspective of passenger flow direction,field theory,and the actual physical significance. The source and the gathering point of passenger flow are shown in Table 1.

**Table 1:** The interpretation of the source point and the gathering point.

Node Type	The Interpretation of Field Theory	The Actual Physical Significance
The Source Point	Passenger flow go from the source point to all directions along radial straight lines.	Subway stations with residential area around in morning rush hours and with offices around in evening rush hours.
The Gathering point	Passenger flow go from all directions to the gathering point along radial straight lines.	Subway stations with residential area around in evening rush hours and with offices around in morning rush hours.

The source point within the stations serves as the main source of passenger flow, the size of which will determine the strength of gravity field in a direct way. Also, the distribution of the gathering points will directly determine the direction, scale and space distribution of passenger flow. The distribution characteristics and field space scale of passenger flow are what arise from the interaction between the source and gathering points.

### 2. Passenger flow field and its characteristic quantity

#### 2.1 The concept of passenger flow field

Field in “Cihai” is defined “The field of physics is an interactive existence of substance in the whole space.” The field has the following basic elements [42]. Firstly,it has a specific zone. Secondly,many elements,here referred to the physical quantity,are in this zone. Thirdly, all the elements,with their own specific distribution laws,interact with each other.

**Definition 5 The gravity field of passenger flow:** Passenger flow refers to how passengers move in a space zone. The gravity field of passenger flow means a space field formed in a space zone with all kinds of elements (including flow and direction) interacting each other. It is like the physical field and established with stations as the source and gathering points of passenger flow, and all the operation lines as the track of movement.

All the elements in the gravity field of passenger flow change with the time. This gravity field is a multidimensional space and a vector field composed of such variables as time and space.

#### 2.2 The field strength of the gravity field of passenger flow

The attraction of passenger flow regarding the given station in URT is related to the size of passenger flow, topological attribute, and its distance away from other stations,i.e.,the acting force received by any point (any other station) in the field is determined by both the source point of *v*_*o*_ and the position of force-beating point of *v*_*i*_.

**Definition 6 The field strength of passenger flow:** The field strength of passenger flow is used to describe the size of deliverability from the source point to any point in the field at a given time.

The field strength is the function of position,time and self-attributes. The positive field strength from the source point of *v*_*i*_ within the distance of *r*_*oi*_ is ${\vec{E}}_{oi}{}^{+}(t)$. Similarly,the negative field strength from the gathering point of *v*_*i*_ within the distance of *r*_*oi*_ is ${\vec{E}}_{oi}{}^{-}(t)$. Node type of passenger flow is shown in Table 1. The expression of the field strength is as follows.

$${\vec{E}}_{oi}{}^{+}(t)=\tau_{1}\cdot \frac{\left\{I_{o}(t)+T_{o}{}^{\mathrm{in}}(t)\right\}\cdot \lambda^{r_{oi}}}{e^{\left(\frac{\beta_{1}}{D}\right)\lambda }\cdot r_{oi}!}\cdot \left(\beta_{2}\cdot BC_{o}{}^{2}\right)\vec{u}$$ (4)

$${\vec{E}}_{oi}{}^{-}(t)=\tau_{2}\cdot \frac{\left\{E_{o}(t)+T_{o}{}^{\mathrm{out}}(t)\right\}\cdot \lambda^{r_{oi}}}{e^{\left(\frac{\beta_{1}}{D}\right)\lambda }\cdot r_{oi}!}\cdot \left(\beta_{2}\cdot BC_{o}{}^{2}\right)\vec{u}$$ (5)

Here *I*_*o*_(*t*), *E*_*o*_(*t*), $T_{o}^{\mathrm{in}}(t)$, and $T_{o}^{\mathrm{out}}(t)$ respectively means incoming passenger flow of station *v*_*o*_, passenger flow leaving station *v*_*o*_, passenger flow of transfer-in and passenger flow of transfer-out of station *v*_*o*_.

*D* is the longest diameter distance of the URT network.
*β*_1_,*β*_2_ is shape parameters to be determined by the scatter diagrams of actual data.
*BC*_*o*_ is the betweenness centrality of the optimum path regarding *v*_*o*_, see Definition 3.
*r*_*oi*_ is the distance between *v*_*o*_ and *v*_*i*_ in the optimum path.
*λ* is the average traveling distance acquired by the actual historical data.
*τ*_1_,*τ*_2_ are the adjusting factors.
$\vec{u}$ is the unit vector of direction.

All the stations attract and diverge passenger flow at the same time. At the force-bearing point of *v*_*P*_, set ${\vec{E}}^{}{}_{iP}(t)$ as the field strength of source point *v*_*i*_, and the total field strength of all the source points at *v*_*P*_ is the scalar sum with regard to every source point. In the meantime, the field strength of passenger flow follows the principle of superposition in the physical field. To describe this relation,the positive matrix of field strength $M_{\vec{E}}^{+}$ is as follows.

$$M_{\vec{E}}^{+}=\left[\begin{array}{cccc} 0 & {\vec{E}}_{12}^{+}(t) & \cdots & {\vec{E}}_{1n}^{+}(t)\\ {\vec{E}}_{21}^{+}(t) & 0 & \cdots & {\vec{E}}_{2n}^{+}(t)\\ \vdots & \vdots & 0 & \vdots \\ {\vec{E}}_{n1}^{+}(t) & {\vec{E}}_{n2}^{+}(t) & \cdots & 0 \end{array}\right]$$ (6)

The sum of column vector in a matrix is the total positive field strength at Station *v*_*P*_. ${\vec{E}}_{P}^{+}(t)={\vec{E}}_{1P}^{+}(t)+{\vec{E}}_{2P}^{+}(t)+\cdots =\sum {\vec{E}}_{iP}^{+}(t)$. Similarly,the negative matrix of field strength is $M_{\vec{E}}^{-}$.

#### 2.3 The potential energy of the gravity field of passenger flow

**Fig 2:**
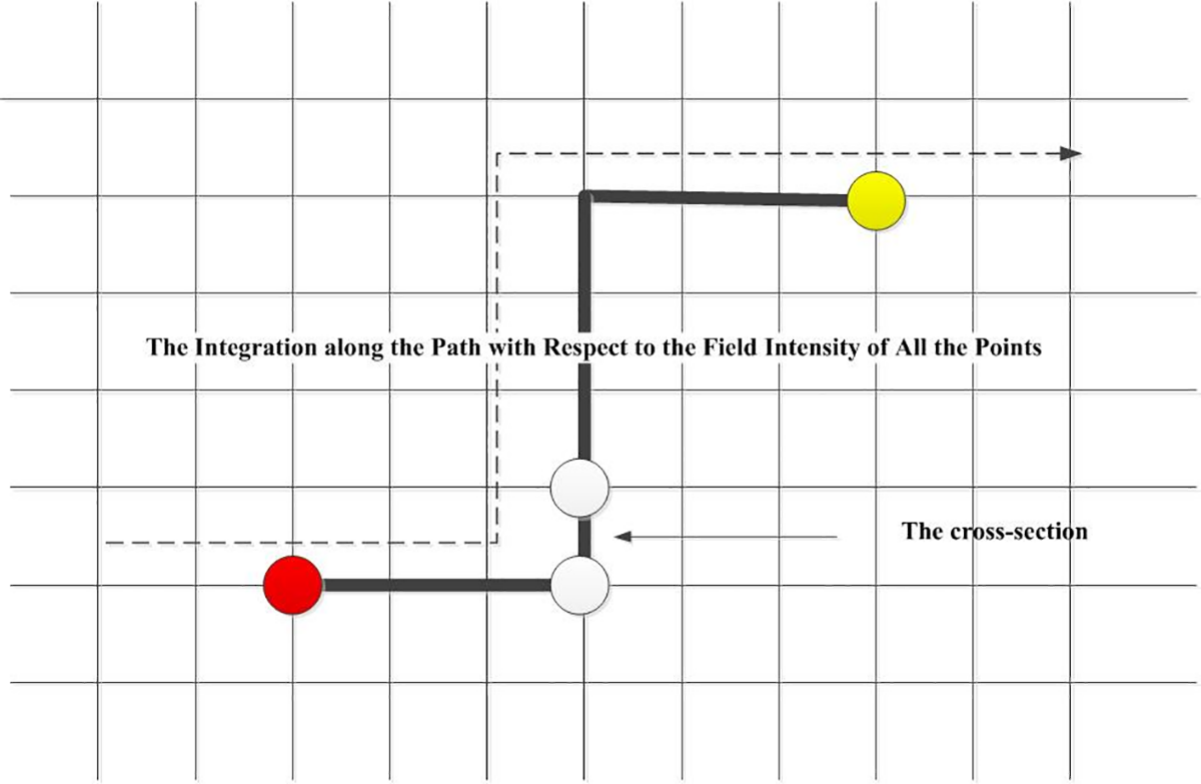
The diagram of the calculation of the potential energy.

**Definition 7 The field potential energy of passenger flow:** The potential energy function means the function of energy aggregation. In other words, it is the circulation capacity of passenger flow between the source point and any point in the field. It is used to describe the scalar of work regarding the field force. Regarding the passenger flow, there is a motional tendency of movement from a high potential energy position to a lower one. The potential energy is the definite integral function of the field strength along the path,see Fig 2.

Here is the expression of potential energy,as follows.

$$\psi_{oi}=-\int_{0}^{T}\int_{0}^{r_{oi}}\vec{E}drdT=-\int_{0}^{T}\int_{0}^{r_{oi}}\sum_{j=1}^{N}{\vec{E}}^{}{}_{jP}(t)drdT$$ (7)

There are many source points in the network. Set *ψ*_*oi*_ as the potential energy of the points *v*_*i*_ and *v*_*o*_, the positive matrix of field energy $M_{\psi_{oi}}^{+}$ are as follows.

$$M_{\psi_{oi}}^{+}=\left[\begin{array}{cccc} 0 & \psi^{+}{}_{12} & \cdots & \psi^{+}{}_{1n}\\ \psi^{+}{}_{21} & 0 & \cdots & \psi^{+}{}_{2n}\\ \vdots & \vdots & 0 & \vdots \\ \psi^{+}{}_{n1} & \psi^{+}{}_{n2} & \cdots & 0 \end{array}\right]$$ (8)

The matrix $M_{\psi_{oi}}^{+}$ falls into the symmetry type, i.e., *ψ*^+^_12_ = *ψ*^+^_21_. In accordance with the principle of the potential energy of physical field,the potential energy at any point *v*_*P*_ is equal to the algebraic sum of potential energy in an independent way, i.e., the sum of column vector regarding the matrix. So is the negative matrix of potential energy $M_{\psi_{oi}}^{-}$.

### 3. The computation of the potential energy

As the function of potential energy is definite integral of field strength, the function of field strength of passenger flow is not continuous, has no primary function. Thus,the Formula of Newton-Leibniz cannot be applied here. The formula of mechanical quadrature of $-\sum_{k=0}^{n}A_{k}\vec{E}(r_{k})$ is adopted in this paper to approximate the value of definite integral.

As for the solution to the potential energy function, the following computation Condition 1 and Condition 2 must be met, both of which are equivalent.

**Condition 1:** In the quadrature formula of the potential energy, $\vec{E}(r_{k})={\tilde{E}}_{k}+\delta_{k}$ is recorded, with *δ*_*k*_ as the error. As for any small positive number *ε* > 0, and in the case of ∃*δ* > 0, if $\left|\vec{E}(r_{k})-{\tilde{E}}_{k}\right|\leq \delta (k=0,1,\cdots ,n)$, then $\left|\sum_{k=0}^{n}A_{k}\left[\vec{E}(r_{k})-{\tilde{E}}_{k}\right]\right|\leq \varepsilon$, with the stabilization of the quadrature formula.

**Condition 2:** In the case of coefficient *A*_*k*_ > 0 (*k* = 0,1,⋯,*n*) in the approximate sequence of number, the approximate formula is stable.

The following is a simple verification.

With any small positive number *ε* > 0 chosen,and in the case of $\delta =\frac{\varepsilon }{r_{oi}-1}$, $\left|\vec{E}(r_{k})-{\tilde{E}}_{k}\right|\leq \delta$ is involved in all *k* = 0,1,⋯,*n*, with

$$\left|\sum_{k=0}^{n}A_{k}\left[\vec{E}(r_{k})-{\tilde{E}}_{k}\right]\right|\leq \sum_{k=0}^{n}\left|A_{k}\right|\left|\vec{E}(r_{k})-{\tilde{E}}_{k}\right|\leq \delta \sum_{k=0}^{n}A_{k}=\delta (r_{oi}-1)=\varepsilon$$ (9)

Making the integral interval of [0,*r*_*oi*_] into *n* equal divisions and in accordance with Condition 2, the Formula of Newton-Cotes is not stable in the case of exponent number *n* ≥ 8. In the URT network model,there are usually at least 8 stations in every line, making the Formula of Newton-Cotes inapplicable.

For improving the computation accuracy of quadrature, we have adopted Composite Simpson's Rule. *n* divisions are made in the interval of [0,*r*_*oi*_], with the Simpson Formula adopted in every sub-interval and the following expressions are acquired.

$$\begin{array}{l} \psi_{oi}=-\sum_{k=0}^{n-1}\int_{r_{k}}^{r_{k+1}}\vec{E}dr\approx S_{n}\\ S_{n}=\frac{h}{6}[\vec{E}(0)+4\sum_{k=0}^{n-1}\vec{E}(r_{k+1/2})+2\sum_{k=0}^{n-1}\vec{E}(r_{k})+\vec{E}(r_{oi})] \end{array}$$ (10)

Since we cannot acquire the primary function,we calculate this quadrature according to the list of one-to-one projection value regarding the existing field strength value and distance in this paper with a classified discussion of *n*.

1. If *r*_*oi*_ is an even number,we will have $n=\frac{r_{oi}}{2}$. With *r*_*k*+1/2_ as an integral number in the computation process,the list of one-to-one projection value regarding the field strength and distance can be employed to get $\vec{E}(r_{k+1/2})$.
2. If *r*_*oi*_ is an odd number,we will have $n=\frac{r_{oi}-1}{2}$. With *r*_*k*+1/2_ not as an integral number in the computation process,we will,with the estimation of polynomial interpolating fitting, acquire $\vec{E}(r_{k+1/2})$ according to the list of one-to-one projection value regarding the existing field strength.

## Data analysis

### 1.The source and background of the data

Here we use passenger flow data of No. 10 Line of Beijing Subway in time of 07:00~08:00 in October 2013 for an analysis and discussion, see **Table 2**.

**Table 2:** The data of passenger flow of Line 10 of Beijing subway.

Station Number	Incoming passenger flow of station	Passenger flow leaving the station	Passenger flow of transfer-in	Passenger flow of transfer-out
L10S0	506	984	----	----
TS/L10S1/L4S6	170	229	2577	3088
L10S2	252	360	----	----
TS/L13S2/L10S3	402	300	2629	1239
L10S4	535	1283	----	----
L10S5	825	815	----	----
L10S6	687	582	----	----
TS/L10S7/L8S9	313	355	1247	1324
L10S8	313	458	----	----
TS/L10S9/L5S8	493	647	4295	2508
TS/L13S12/L10S10	691	585	2682	1713
L10S11	867	622	----	----
TS/AES1/L10S12	1696	1858	0	0
L10S13	1125	1248	----	----
L10S14	272	340	----	----
L10S15	816	1238	----	----
TS/L10S16/L6S12	272	401	2845	2137
L10S17	142	610	----	----
TS/L1S19/L10S18	1065	630	3067	2737
L10S19	1303	962	----	----
L10S20	2200	1395	----	----
L10S21	994	613	----	----
L10S22	1791	868	----	----
L10S23	2443	318	----	----
L10S24	933	331	----	----
TS/YLS0/L5S22	864	388	3225	3512
L10S26	878	312	----	----
L10S27	1188	1595	----	----
L10S28	809	374	----	----
TS/L10S29/L4S22	377	169	3857	4605
L10S30	544	311	----	----
L10S31	307	126	----	----
L10S32	896	257	----	----
L10S33	477	126	----	----
L10S34	479	291	----	----
TS/L14S6/L10S35	369	183	1145	1446
TS/L10S36/L9S6	773	628	3411	2683
L10S37	239	216	----	----
TS/L1S7/L10S38	461	774	3232	2621
L10S39	494	730	----	----
TS/L10S40/L6S1	195	130	610	659
L10S41	412	308	----	----
L10S42	313	626	----	----
L10S43	221	137	----	----
L10S44	236	442	----	----

**Fig 3:**
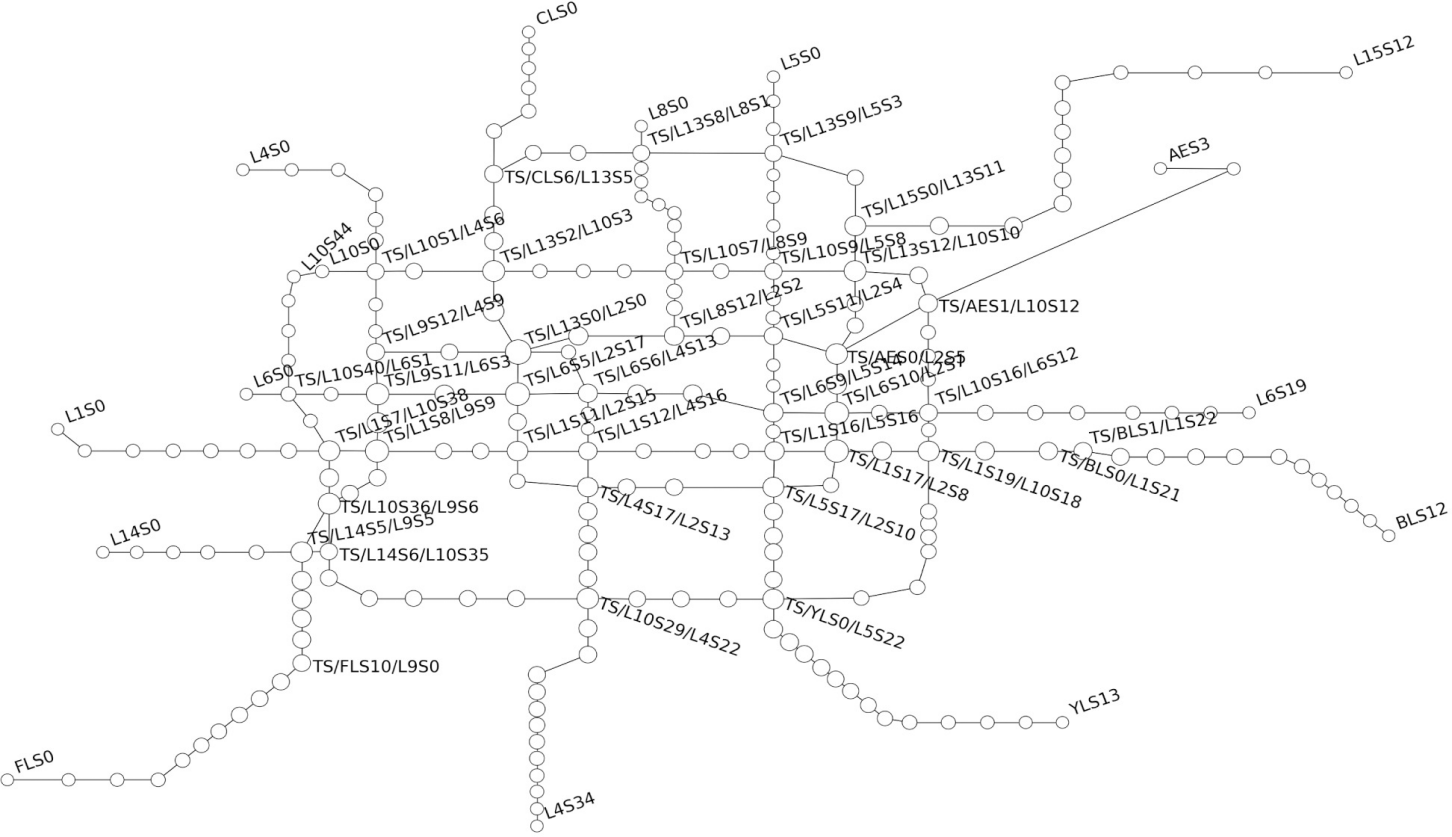
The topological diagram of operation network regarding Beijing rail transit.

The construction of the topological diagram of Beijing Subway is shown in Fig 3.

### 2. The verification of the parameters of characteristic quantity

If the gravity field of passenger flow is composed of one single line, the parameter of *λ* is the average number of travel stations along this line. If we take the whole URT network as the passenger flow field, the parameter of *λ* is the average number of travel stations in the network. The case study and background in this paper are aimed at the topological diagram of the whole network in Beijing URT, with the choice of *λ* = 11.

At the same time, the values of the parameter of *D* in the formula of the field of passenger flow are composed of two parts. Firstly, for passenger flow with one traveling line, *D* is the total length of this line. Secondly, in regard to passenger flow of transfer, *D* is the maximum diameter distance in the network.

**Fig 4:**
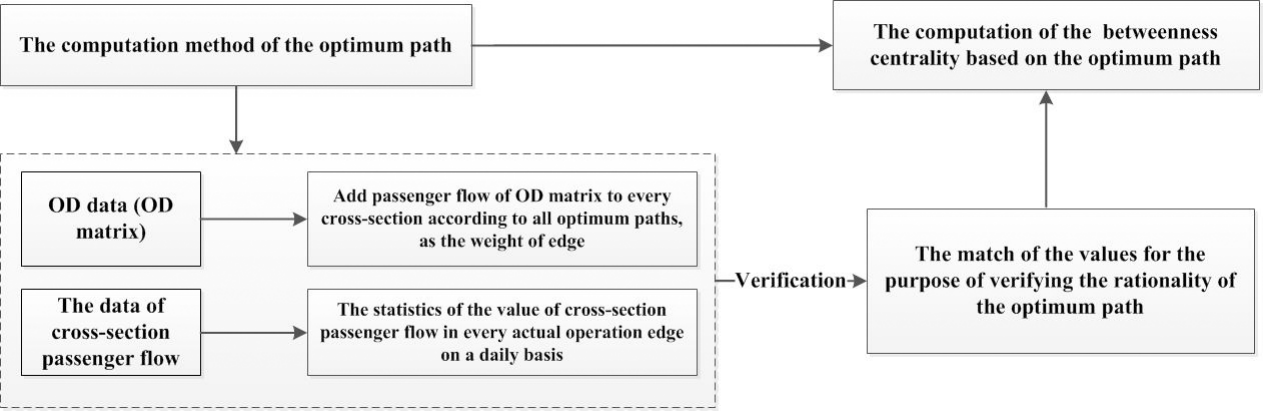
The verification method of the optimum path of passenger flow.

According to Definition 2, we have firstly conducted a verification of the optimum path based on passenger flow data, the method of which is shown in **Fig 4**.

With every cross-section passenger flow introduced into the network and based on the OD matrix, we weight every edge according to the optimum path. And it turns out when *κ* = 1.531 the model has the best effect, get more details in chapter 5 of Reference [43].

### 3. The force analysis of the spatial linkage of passenger flow regarding the URT network

#### 3.1 The “force” analysis of field strength

**Fig 5:**
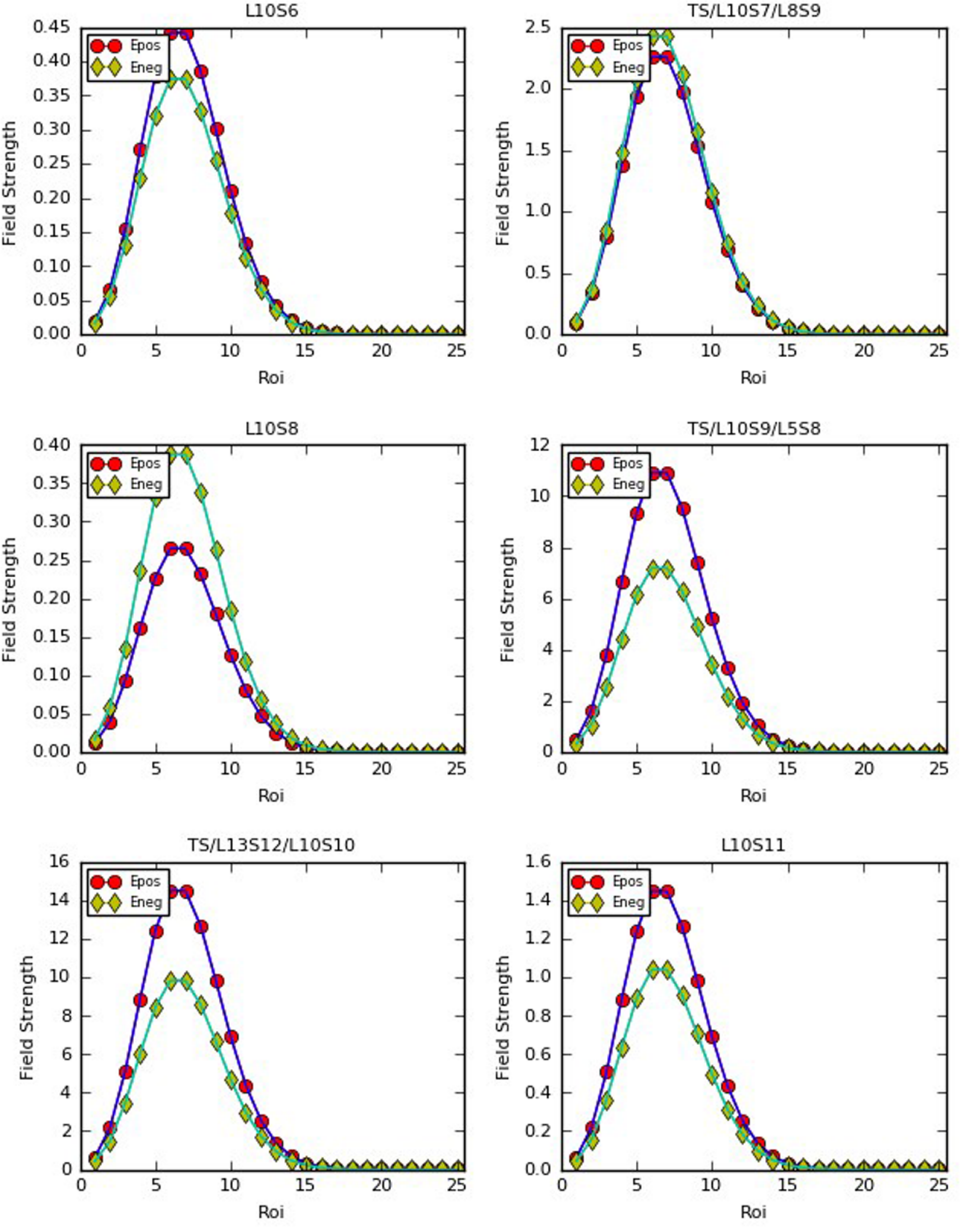
The positive and negative field strength of the source points regarding six stations in No. 10 Line of Beijing subway.

With some stations at Line 10 as the source (gathering) points of passenger flow, the following is the diagrammatic sketches of positive and negative field strength that changes with the different distance of *R*_*oi*_ at the six typical stations, see Fig 5.

The analysis has established that, the size of field strength is directly related to the average traveling stations, the incoming passenger flow and leaving out of station and the betweenness centrality of the node. A stronger strength will lead to a bigger attraction of passenger flow between two stations. The following can be known from Fig 5. Firstly, taking Zhichunlu Station (TS/L13S2/L10S3) and Zhichunli Station(L10S2) for example, as a transfer station, Zhichunlu Station has a bigger load of passenger flow, and a larger betweenness centrality value, so its values of positive and negative field strength obviously exceed Zhichunli Station. Secondly, as the average traveling distance is *λ* = 7 at No. 10 Line, the field strength of *E*_max_ turns out to be at the station whose distance is *R*_*oi*_ = 7 from the source point; passengers are more likely to get off at this station from the perspective of probability.

**Fig 6:**
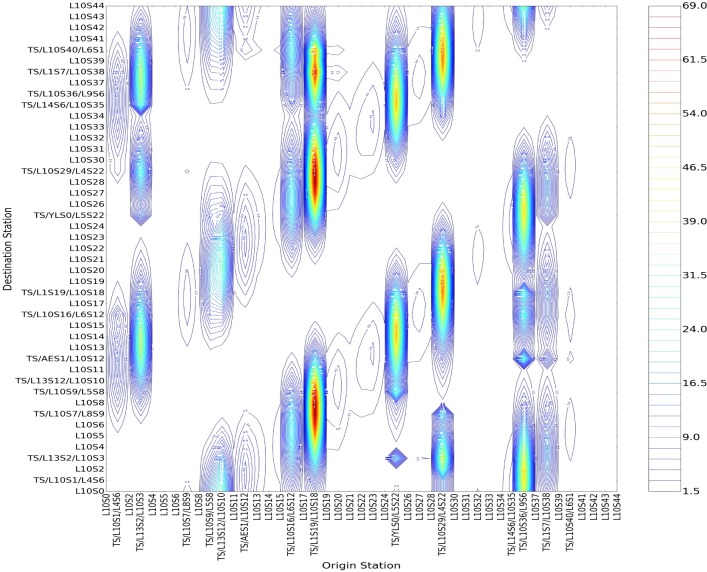
The contour lines of positive field strength of the source points regarding all stations of No. 10 Line of Beijing subway.

**Fig 7:**
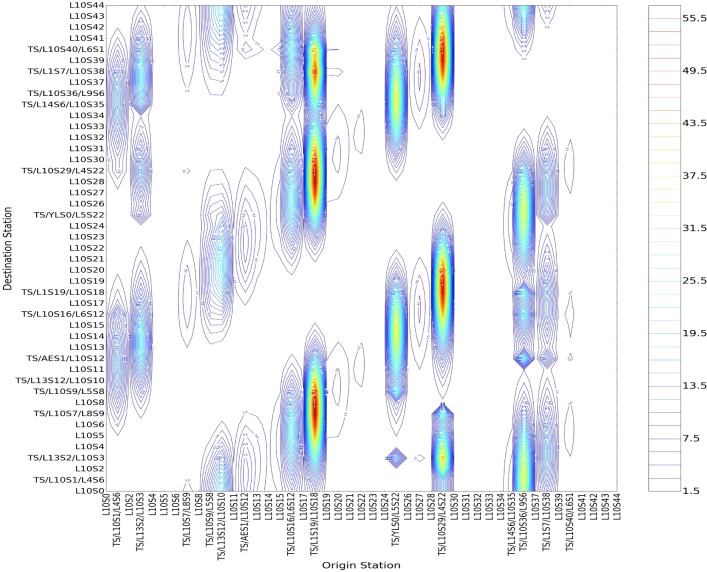
The contour lines of negative field strength of the gathering points regarding all stations of No. 10 Line of Beijing subway.

We can get the following conclusions after an analysis of Figs 6 and 7. Firstly, as for different stations with the same size of incoming passenger flow, a bigger betweenness centrality will lead to stronger field strength. Taking Shiliuzhuang (L10S26) and Jiaomendong (L10S28) as example, the betweenness centrality of the L10S26 is 0.09704 and L10S28 is 0.09655, and the field strength of the former evidently exceeds the latter with the same incoming and leaving-out size. Secondly, regarding the same stations, different incoming size will lead to different positive and negative field strength. Taking the transfer station of Zhichunlu(TS/L13S2/L10S3) as an example, the transfer-in size into No. 10 Line from other lines (No. 13 Line to No. 10 Line) obviously exceeds the transfer-out size (No. 10 Line to No. 13 Line), showing that the value of positive field strength is bigger than that of negative field strength, i.e., the divergence capacity of passenger flow at Zhichunlu Station is stronger than its convergence capacity. Thirdly, the transfer stations have a larger load of passenger flow in the whole line, and their betweenness centrality are bigger than those of ordinary stations. Therefore, the stations with the biggest positive field strength are as follows in a descending order, i.e., Guomao (TS/L1S19/L10S18), Jiaomenxi (TS/L10S29/L4S22), Songjiazhuang (TS/YLS0/L5S22), Liuliqiao(TS/L10S36/L9S6), Zhichunlu (TS/L13S2/L10S3) and Shaoyaoju (TS/L13S12/L10S10). The stations with the biggest negative field strength are as follows in a descending order, i.e., Guomao (TS/L1S19/L10S18), Jiaomenxi (TS/L10S29/L4S22), Songjiazhuang (TS/YLS0/L5S22), Liuliqiao(TS/L10S36/L9S6). Lastly, the blank zones in the Figs 6 and 7 are the zones with an exceedingly tiny value of field strength that can be ignored and regarded as zero, showing a relatively small attraction of passenger flow among these zones.

The following is an analysis of a stronger attraction of passenger flow, i.e., the zones with relatively big positive field strength. Taking Guomao Station(TS/L1S19/L10S18) as an example, the passengers entering in Guomao Station are more likely to get off at the zones in Table 3.

**Table 3:** The zones of “Jintaixizhao-Guomao” with bigger values of positive field strength.

Serial Number	Zones with the Biggest Positive Field Strength	The Actual Physical Significance
Zone 1	TS/L10S36/L9S6-TS/L10S40/L6S1	Liuliqiao Station to Cishousi Station
Zone 2	TS/YLS0/L5S22- L10S31	Songjiazhuang Station to Jijiamiao Station
Zone 3	L10S5-TS/L13S12/L10S10	Mudanqiao Station to Sanyuanqiao Station

A denser contour line of field strength will lead to greater changes in its value, with the biggest value in the central point. Greater field strength means a stronger attraction of passenger flow between two stations, with a relatively big probability of existence of the largest cross-section passenger flow in the path. With an analysis of several typical zones in the table and the comparison of the actual traveling data and that of cross-section passenger flow, we can establish that passengers in those zones are more likely to get off at Guomao Station in morning rush hours.

#### 3.2 The “potential” analysis of the potential energy

**Fig 8:**
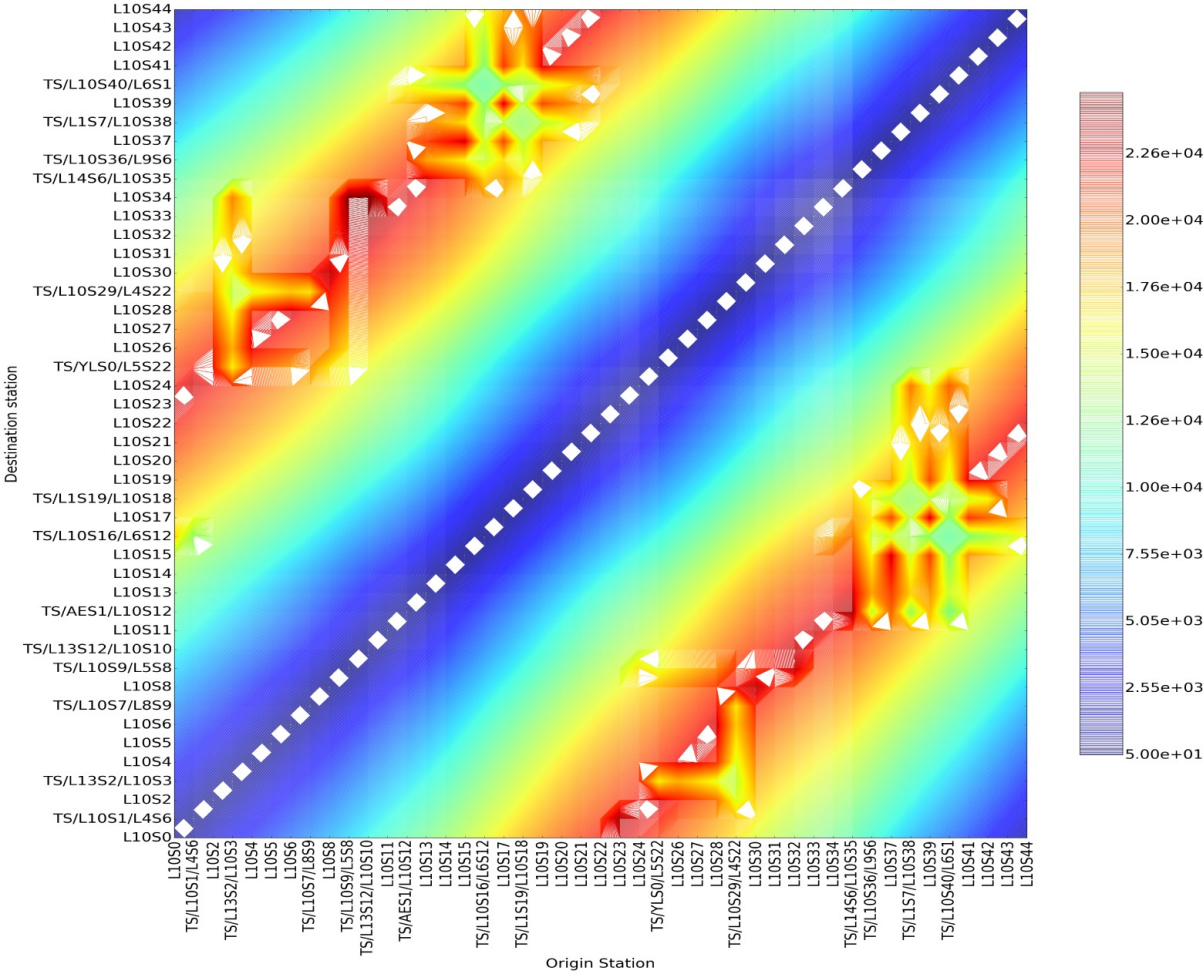
The contour lines of potential energy with all stations of No. 10 Line of Beijing subway as the source points.

Taking every station of Line 10 as the source point of passenger flow, the value of potential energy at any point of Line 10 can be calculated in accordance with the integral of the field strength along the optimum path. The value of total field strength in every point is the summarization of column vector of matrix, so the results of potential energy based on the positive and negative field strength are almost equal. The contour lines of potential energy based on positive field strength are shown in **Fig 8**.

We know that the potential energy describes the aggregation energy of passenger flow at any point in the field.

An analysis of the diagram of contour lines has helped us conclude that passenger flow and its aggregation capacity in the two zones of high potential energy will reach the maximum value. What’s more, as the potential energy is the integral of the field strength along the path, a longer path between the two zones will lead to bigger potential energy. Here is a description of several typical couple zones of potential energy.

1. According to Fig 8, there are seven typical couple zones of high potential energy in Line 10, i.e., are shown in **Table 4**. A longer path with zones of higher field strength between two zones in the network means a stronger attraction of passenger flow. For example, we have the zones of high field strength of “Mudanyuan-Sanyuanqiao” and “Songjiazhuang-Jijiamiao”. The analysis of the actual physic al significance is as follows. Firstly, from the perspective of incoming passengers, some big stations with a big incoming size are at the main path of passenger flow, e.g., Dawanglu, Sanyuanqiao, Songjiazhuang, and Jiaomenxi Station. The gathering capacity of the passenger flow during this time interval reaches the maximum, which verifies the definition of high potential energy. Secondly, in combination with the actual data of cross-section passenger flow, several high sections all exist at the main optimum path, e.g., Shuangjing-Guomao Section, Jintaixizhao -Hujialou Section, Zhichunli-Zhichunlu Section, which verifies the actual significance of zones of high potential energy.
2. There are also some couple zones of low potential energy in the Line 10, i.e., such as Suzhoujie Station (L10S0) to Zhichunlu Station(TS/L13S2/L10S3) and Chedaogou Station (L10S41) to Bagou Station(L10S44) and so on in Fig 8. A shorter path means a weaker aggregation capacity for passenger flow, with the consequently smallest potential energy among the neighborhoods, resulting in the “couple zones” of low potential energy.

**Table 4:** The zones of high potential energy in the line 10.

Serial Number	Couple Zones of the highest potential energy	The Actual Physical Significance
Couple Zone 1	“L10S0-L10S2” and “L10S21-L10S26”	“Suzhoujie-Zhichunli” and “Panjiayuan-Shiliuzhuang”
Couple Zone 2	TS/L13S2/L10S3 and “L10S23-L10S24”	“Zhichunlu Station” and “Fenzhongsi-Chengshousi”
Couple Zone 3	“L10S4-L10S6” and “L10S26-L10S28”	“Xitucheng-Jiandemen” and “Shiliuzhuang-Jiaomendong”
Couple Zone 4	“TS/L10S7/L8S9-L10S8” and “L10S27-L10S31”	“Beitucheng-Anzhenmen” and “Dahongmen-Jijiamiao”
Couple Zone 5	“TS/L10S9/L5S8-L10S11” and “TS/L10S29/L4S22-TS/L10S36/L9S6”	“Huixinxijienankou-Taiyanggong” and “Jiaomenxi-Liuliqiao”
Couple Zone 6	“TS/AES1/L10S12-L10S14” and L10S32-TS/L10S36/L9S6	“Sanyuanqiao-Nongyezhanlanguan” and “Shoujingmao-Liuliqiao”
Couple Zone 7	“L10S19-L10S22” and “L10S41-L10S44”	“Shuangjing-Shilihe” and “Chedaogou-Bagou”

## Conclusions

Based on the construction of URT network and regarding the field theory, we have conducted a quantitative analysis of passenger flow with regard to space-time distribution and its potential characteristics in the network. With the concept of the gravity field of passenger flow, the field strength and potential energy are proposed. The key parameters have been offered, including the definition of betweenness centrality based on the optimum path, and the employment of Composite Simpson's Rule.

With a simulation and verification of the proposed model, there are some results. Firstly, the analysis of **“force”** helps us know that a denser field strength means great changes in its values, with that the center of field strength contour reaching relative maximum value and a high probability of the largest section of passenger flow. For the stations of high field strength, passengers who entering in these stations are more likely to get off at Guomao Station, because it is a typical working zone with a strong attraction of passenger flow at the morning rush hours. Secondly, the analysis of the **“potential”** shows that the interval of two zones with high potential energy will have the maximum transfer capacity. In combination with the actual data of cross-section passenger flow, high sections of passenger flow in the rush hours exist in the main circulation path.

Although this paper has achieved some meaningful results, some parameters of the proposed model is still need to be improved in the future. Firstly, we will improve the computation of potential energy by adding the influence factor of passenger flow changes. Secondly, Gamma function, the continuation of factorial, can be employed to error estimation in factorial of potential energy.

## Supporting information

S1 File Passenger flow data. (XLSX)
